# Abdominal Tuberculosis Complicated by Intestinal Perforation

**DOI:** 10.1155/2021/8861444

**Published:** 2021-02-09

**Authors:** Michiel L. Sala, Samuel M. Verhage, Frank Zijta

**Affiliations:** ^1^Department of Radiology, Haaglanden Medical Center, The Hague, Netherlands; ^2^Department of Surgery, Haaglanden Medical Center, The Hague, Netherlands

## Abstract

Although relatively rare, there is an increasing incidence of abdominal tuberculosis (TB) in the developed countries, with the peritoneum being the most common site of involvement. Manifestation of abdominal TB should be considered in patients with relevant clinical symptoms and risk factors, including a history of prior TB infection and residence in or travel to an area where tuberculosis is endemic. We report a case of intestinal tuberculosis with a complicated disease course after the completion of treatment. Persisting abdominal symptoms during or after treatment should raise suspicion of subclinical intestinal obstruction. Early clinical recognition and surgical treatment may avoid poor outcome due to intestinal perforation.

## 1. Case Report

**Figure 1:**
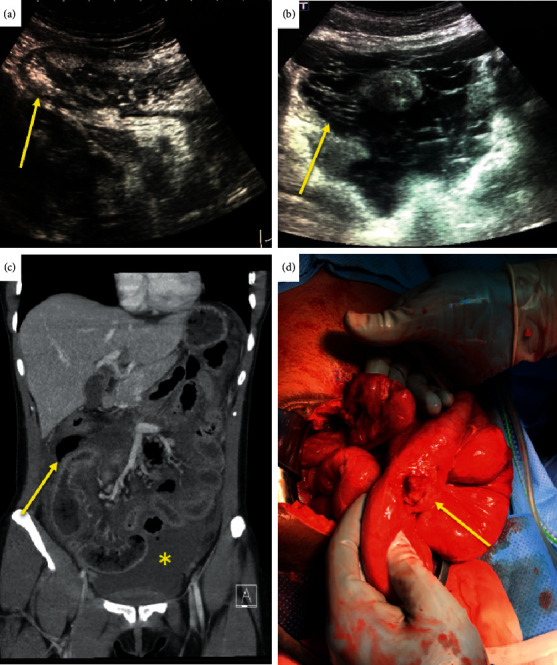
Ultrasound imaging reveals submucosal ileal wall thickening (a) (arrow) and free and loculated ascites (b) (arrow). Subsequent contrast-enhanced abdominal computed tomography showing ascites (asterix) and free air (arrow) in the peritoneal cavity in close relation to affected terminal ileum wall (c). Laparotomy showed many adhesions and focal omental thickening adhering to a thickened distal ileum loop cavity (d) (arrow).

A 32-year-old woman presented with progressive generalized abdominal pain, nausea, and vomiting. She recently travelled from India where she had completed a 6-month antituberculous therapy for intestinal tuberculosis (TB) diagnosed with endoscopy in India. On physical examination she was found to be severely ill with abdominal distension and generalized tenderness, with no guarding. Vital signs were as follows: heart rate, 127 beats per minute; blood pressure, 102/54 mm Hg; and temperature, 37.6 degree Celsius. Inflammatory markers were markedly raised with increased white blood cell count (23.1*∗*10^9^/L, reference range 4.0–10.0*∗*10^9^/L) and C-reactive protein (210 mg/L, reference range 0–8 mg/L) levels. Ultrasound imaging revealed free and multiloculated ascites (Figure 1). Ultrasound-guided fine-needle aspiration of the loculated ascites was performed, with aspiration of cloudy yellowish fluid which was sent for cytopathologic and medical microbiology examination. Chest X-ray showed no signs of pulmonary TB. Subsequent contrast-enhanced abdominal computed tomography showed ascites, diffuse small bowel wall thickening with subsequent enhancement, and enhanced peritoneal thickening. In addition, free air in the peritoneal cavity was detected which was distributed in close relation with the terminal ileum (Figure 1). In context with the past medical history and clinical presentation, a working diagnosis of tuberculous peritonitis with small bowel perforation was made. It was decided to perform an emergency laparotomy with patient approval. Accordingly, many adhesions and focal omental thickening adhering to a thickened distal ileum loop were found (Figure 1). A microperforation and a manifest stenosis were found in the terminal ileum. A fecal peritonitis was not found. Resection of approximately 20 centimeter of the terminal ileum was performed followed by stapled side-to side anastomosis because of the microperforation and a manifest stenosis. Inspection of the residual small and short bowel showed no signs of stenosis. Histopathology revealed multifocal transmural inflammation and fibrinous exudate over the peritoneal surface of the resected ileal specimen, but no evident granulomas. Polymerase chain reaction and Ziehl–Neelsen and Auramine staining were negative for TB. In addition, definite cultures for *Mycobacterium tuberculosis* were negative.

In context with the past medical history and clinical presentation, a diagnosis of ileal perforation after completion of antituberculous treatment for intestinal tuberculosis with concurrent tuberculous peritonitis was made. The patient was discharged 6 days postadmission in good clinical condition after completing antibiotic treatment with cefuroxime and metronidazole. On follow-up, she reported satisfactory recovery.

## 2. Discussion

Immigrants from developing countries where tuberculosis is endemic are at increased risk for abdominal TB [1]. In addition, human immunodeficiency virus, acquired immunodeficiency syndrome, alcoholism, intravenous drug use, steroid therapy, and elderly age are also established risk factors for abdominal tuberculosis [1]. The peritoneum is most commonly involved in abdominal tuberculosis. Tuberculous peritonitis usually affects young adults but may occur at any age. The disease is thought to occur following reactivation of latent tuberculous foci in the peritoneum, via hematogenous spread from a previous pulmonary infection [2]. TB peritonitis can also occur via ingestion of mycobacteria, for example, by ingestion of unpasteurized milk, with passage through Peyer's patches in the ileum region to mesenteric lymph nodes. Contiguous spread from ileocecal TB is another route of possible infection. Much less commonly, direct spread from tuberculous salpingitis or hematogenous spread from active pulmonary infection may occur [2]. Patient symptoms frequently include abdominal pain and fever. In addition, in the “wet type”TB peritonitis, patients present with a large amount of free or loculated ascites, seen in most patients. The less common fibrotic and the “dry” or “plastic” type may present as diffuse, confluent omental and peritoneal disease, mesenteric lymphadenopathy, and adherent bowel loops [2, 3]. An operation can be very difficult because of a frozen abdomen. Symptoms have frequently persisted for weeks or months before a diagnosis is established. A diagnosis of TB peritonitis should be considered in patients with applicable clinical symptoms and risk factors (e.g., history of prior TB infection and residence in or travel to an area where tuberculosis is endemic). In patients with suspected abdominal tuberculosis, ultrasound imaging should be performed, which may show ascites and echogenic debris, peritoneal and omental thickening, bowel wall thickening, and lymphadenopathy [3]. Computed tomography (CT) imaging confirms these findings and can be useful in assessing disease dissemination. In addition, abdominal CT can be helpful in distinguishing peritoneal TB, for example, from peritoneal carcinomatosis by showing smooth peritoneal thickening, lymphadenopathy with hypodense centers, and calcifications and manifesting splenic lesions [2]. Importantly, CT is able to assess potential complications arising from inflammation and adhesions, including intestinal obstruction, bowel perforation, abscesses, and fistulae. In these cases, surgical intervention is frequently required.

Differential diagnostic considerations for TB peritonitis include carcinomatosis, ovarian cancer, mesothelioma, or non-TB peritonitis. In addition, end-stage liver disease with coexisting ascites and spontaneous bacterial peritonitis may have similar appearance although signs of chronic liver disease on physical examination and imaging studies are usually present [2, 4]. While a high index of clinical suspicion is required, microbiological or histopathological confirmation is usually required to establish a definitive diagnosis of TB peritonitis. Ultrasound or CT imaging can be used to guide needle aspiration of ascitic fluid or peritoneal biopsy, by which *Mycobacterium* can be demonstrated in an involved site (e.g., peritoneum or intestine). Patients with abdominal TB are treated with antituberculous therapy. Our patient presented with ileal perforation after completion of a 6-month antituberculous therapy. In accordance with this disease course, previous case studies have reported bowel perforation after the completion of antituberculous treatment [5, 6]. Intestinal involvement may include single or multiple transverse, circumferential, transmural ileal, or jejunal ulcers that form strictures during the healing process which may subsequently perforate [5, 6]. After consultation with our patient's treating physician, ileal ulcers were established by endoscopy in her initial diagnostic workup in India. Cicatrization after healing of intestinal ulcers may thus be a possible explanation for the complicated disease course in our patients. An alternative explanation might be a paradoxical response phenomenon, with a paradoxical deterioration of disease after starting medical therapy [4]. Theoretically, a radical decrease in bacterial load stimulates a host delayed type hypersensitivity response with subsequent tissue damage [4]. Otherwise, drug resistance may be a cause of disease progression after finishing treatment. However, in our case, polymerase chain reaction and Ziehl–Neelsen and Auramine staining were negative for TB. In addition, definite cultures for *Mycobacterium tuberculosis* were negative. Finally, we did not find any histological evidence of prevalent granulomas. Therefore, it is plausible that stricture formation with subsequent perforation has occurred in our patient. Persisting abdominal symptoms during or after treatment should raise suspicion of potential (subclinical) intestinal obstruction. Early clinical recognition and surgical treatment may avoid poor outcome of intestinal perforation [4].

## 3. Conclusion

Although relatively rare, there is an increasing incidence of abdominal tuberculosis in the developed countries. We report a case of intestinal tuberculosis complicated by ileal perforation which was treated surgically with concurrent tuberculous peritonitis after the completion of antituberculous treatment. The diagnosis was made in the appropriate clinical context, by using ultrasound and computed tomography imaging and subsequent intraoperative findings. Our patients reported satisfactory recovery on clinical follow-up.

## References

[1] Patel S., Al-Nowfal A., Gould S. T. (2012). A rare cause of faecal peritonitis: jejunal perforation in a patient undergoing treatment for pulmonary tuberculosis. *Journal of Surgical Case Reports*.

[2] Vaid U., Kane G. C. (2017). Tuberculous peritonitis. *Microbiology Spectrum*.

[3] Sivrioglu A. K., Incedayi M., Saglam M. (2013). Wet type of tuberculous peritonitis. *BMJ Case Report*.

[4] Lee M. J., Cresswell F. V., John L., Davidson R. N. (2012). Diagnosis and treatment strategies of tuberculous intestinal perforations. *European Journal of Gastroenterology & Hepatology*.

[5] Rathi P., Gambhire P. (2016). Abdominal tuberculosis. *Journal of the Association of Physicians of India*.

[6] Park Y. S., Jun D. W., Kim S. H. (2008). Colonoscopy evaluation after short-term anti-tuberculosis treatment in nonspecific ulcers on the ileocecal area. *World Journal of Gastroenterology*.

